# Concomitant calcaneo-cuboid-cuneiform osteotomies and the modified Kidner procedure for severe flatfoot associated with symptomatic accessory navicular in children and adolescents

**DOI:** 10.1186/s13018-014-0131-2

**Published:** 2014-12-05

**Authors:** Jung Ryul Kim, Chan Il Park, Young Jae Moon, Sung Il Wang, Keun Sang Kwon

**Affiliations:** Department of Orthopaedics Surgery, Research Institute for Endocrine Sciences and Research Institute of Clinical Medicine, Chonbuk National University Medical School, 567 Baekje-ro, Dukjin-gu, Jeonju 561-756 South Korea; Department of Preventive Medicine, Chonbuk National University Medical School, 567 Baekje-ro, Dukjin-gu, Jeonju 561-756 South Korea

**Keywords:** Flatfoot, Accessory navicular, Calcaneo-cuboid-cuneiform osteotomy, Modified Kidner procedure

## Abstract

**Background:**

Accessory navicular can become symptomatic in childhood, and in some cases, the condition is associated with progressive flattening of the longitudinal arch. Moreover, some severe, rigid flatfoot deformities are associated with an accessory navicular. We investigated the results of concomitant calcaneo-cuboid-cuneiform osteotomies (triple C) and the modified Kidner procedure for severe flatfoot associated with a symptomatic accessory navicular in children and adolescents.

**Methods:**

Twenty-one feet of 13 patients (nine boys, four girls; mean age 12.7 years) with severe flatfoot associated with a symptomatic accessory navicular who were treated with concomitant triple C and the modified Kidner procedure were evaluated based on clinical and radiographic examinations preoperatively and at a mean follow-up of 22.4 months (range, 12–36 months). We measured 12 variables on the anteroposterior (AP) and lateral weight-bearing radiographs, and we used the American College of Foot and Ankle Surgeons (ACFAS) score for clinical assessment.

**Results:**

We found significant improvements (*p* < 0.001) in eight of the 12 radiographic measurements: the AP talo-first metatarsal (MTT) angle, AP talo-navicular coverage angle, AP talo-calcaneal angle, lateral talo-first MTT angle, calcaneal pitch, lateral talo-calcaneal angle, lateral talo-horizontal angle, and naviculo-cuboid overlap. Average ACFAS scores were significantly improved at the time of the last follow-up (*p* < 0.001). The only complication was overcorrection of the hindfoot in one patient.

**Conclusions:**

Concomitant triple C and the modified Kidner procedure result in favorable radiographic and clinical outcomes in the treatment of severe flatfoot associated with a symptomatic accessory navicular in children and adolescents.

## Background

An accessory navicular is a supernumerary ossicle that occurs within the tibialis posterior tendon (TPT) at its navicular insertion. Symptoms develop with recurrent stress at the synchondrosis between the accessory bone and the medial navicular tuberosity secondary to a pull of the TPT or trauma [1]. Kidner originally concluded that the accessory navicular interferes with the normal leverage of the tibialis posterior, causing a weak longitudinal arch and flatfoot [2]. Although subsequent studies have not found a relationship between the accessory navicular and the development of flatfoot [3], an accessory navicular can become symptomatic in childhood, and in some cases, the condition is associated with progressive flattening of the longitudinal arch. Moreover, some severe, rigid flatfoot deformities are associated with an accessory navicular [2,4].

As with most orthopedic conditions, the first line of treatment for an accessory navicular is nonoperative. Operative intervention is indicated for patients who are recalcitrant to nonoperative treatment and have persistent pain and dysfunction. Current operative treatments include simple excision of the accessory bone from within the TPT and the modified Kidner procedure, which involves excision with takedown and reattachment of the TPT (with or without advancement) [5-7]. These techniques give good results for symptomatic accessory navicular. In addition, patients presenting with medial foot pain and prominence should be evaluated for an accessory navicular as the cause of their pain. Although the contribution of a symptomatic accessory navicular to the development of flatfoot deformity remains controversial, it has been shown that treatment of the accessory navicular alone may not correct this deformity. Symptomatic flatfoot deformity can be managed with various soft tissue and bony procedures. A range of surgical options, including soft tissue reconstruction, osteotomy, arthroereisis, and arthrodesis, have been introduced; however, single soft tissue imbrications, arthroereisis, and arthrodesis have unsatisfactory outcomes because they can lead to recurrence of the deformity and adjacent degenerative arthritis [8]. Therefore, many investigators have suggested that joint-sparing procedures, such as lateral column lengthening (LCL) and calcaneo-cuboid-cuneiform osteotomies (triple C), should be the treatment of choice for the correction of flatfoot deformities in children [9-11]. Kim et al. found that the triple C is a more effective procedure than LCL for the correction of severe pediatric flatfoot deformity [12].

In the current study, we assessed the outcomes of combined triple C and the modified Kidner procedure for severe, symptomatic flatfoot associated with an accessory navicular in children and adolescents. We hypothesized that these procedures would lead to improvement of pain, function, and correction of the flatfoot deformity.

## Methods

This study was approved by the institutional review board at our institution. The presence of an accessory navicular was confirmed on plain radiographs, and the diagnosis of flatfoot deformity was based on a poor formation of the arch and a valgus position of the heel during weight bearing. The inclusion criteria were (1) consecutive patients who underwent a combined triple C and modified Kidner procedure for severe flatfoot deformity associated with an accessory navicular between 2006 and 2012, (2) patients who had preoperative and postoperative weight-bearing anteroposterior (AP) and lateral foot radiographs, and (3) patients with a minimum follow-up of 1 year. Patients with severe deformity were those who could not reconstitute the arch and who had a varus position of the heel while on tiptoes. The exclusion criteria were underlying neurological or syndromic conditions, tarsal coalition, or inadequate radiographs for measurement.

During the study period, 66 patients (70 feet) were diagnosed with a symptomatic accessory navicular and underwent operative treatment. Of those patients, 55 had concomitant flatfoot deformity. Twenty-four feet had severe deformity and underwent a combined triple C and modified Kidner procedure. Three feet were lost to follow-up. The other 31 feet were treated with the modified Kidner procedure only. Twenty-one feet of 13 patients were available for follow-up and were included in this study. There were nine boys and four girls with a mean age at the time of surgery of 12.7 (range, 10–16) years. The mean follow-up was 22.4 (range, 12 to 36) months. All were type II accessory navicular. All procedures were performed by a single surgeon (KJR).

### Surgical technique

**Figure 1:**
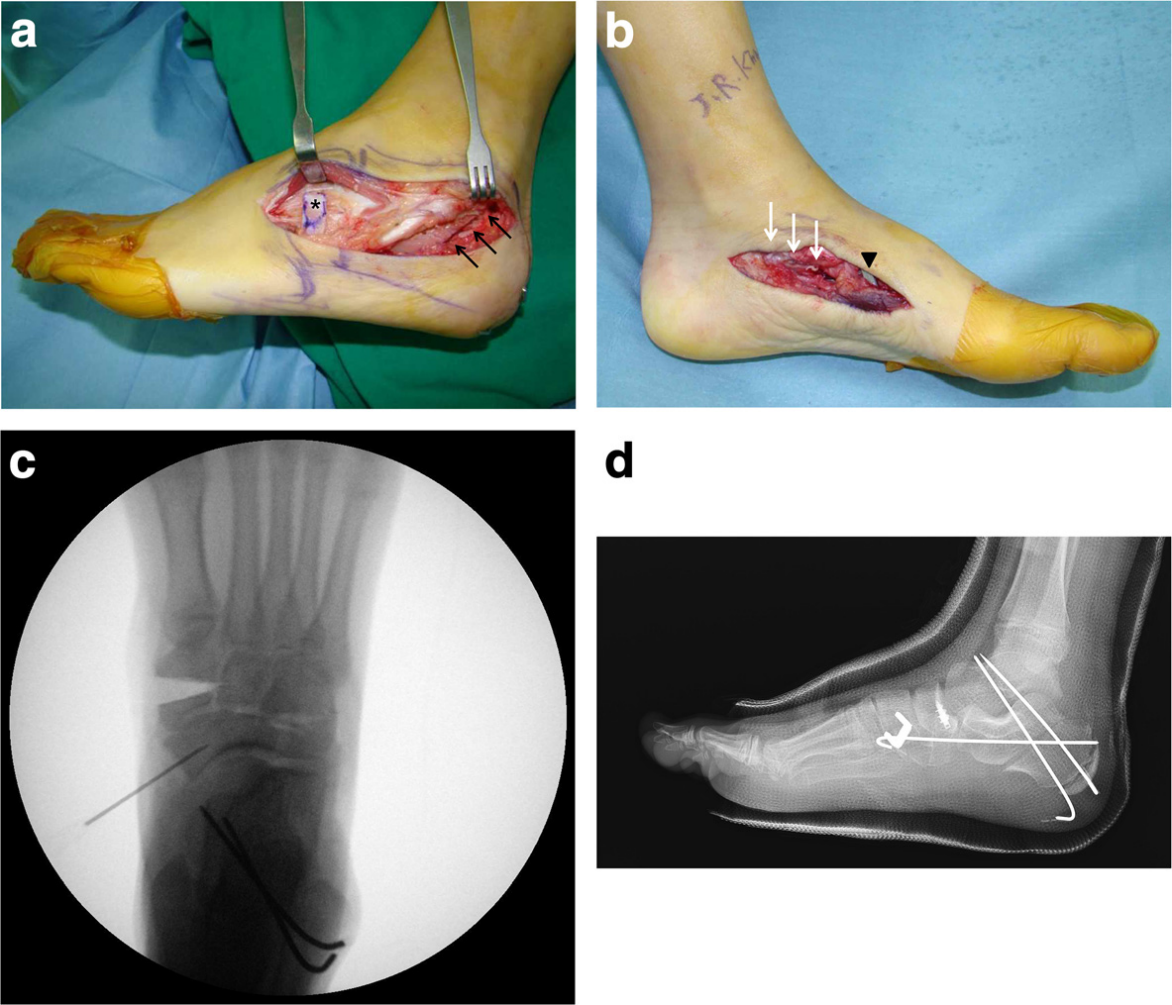
**Triple C combined with the modified Kidner procedure. (a)** Lateral view of the foot showing calcaneal osteotomy (black arrows) and insertion of the opening wedge graft from the cuneiform into the cuboid (asterisk). **(b)** Medial view of the foot showing plantar flexion and pronation of the foot achieved with closing wedge osteotomy of the medial cuneiform (arrowhead), providing reconstruction of the longitudinal arch, and the TPT was advanced and attached to the navicular with the two no. 2 braided nonabsorbable sutures attached to the anchor (white arrows). **(c)** Positioning of the anchor was confirmed under intraoperative fluoroscopy. **(d)** Immediate postoperative radiography after combined triple C and modified Kidner procedure.

The surgical technique for triple C was similar to that reported by Rathjen and Mubarak [8]. The lateral cortex of the calcaneus was cut with an oscillating saw, and the medial cortex of the calcaneus was cut with an osteotome or a rongeur under direct vision. As much as possible, the osteotomy was made parallel to the posterior facet of the subtalar joint. The weight-bearing portion of the calcaneus was displaced by at least one half of the width of the calcaneus. A smooth Steinmann pin was drilled from the posterior aspect of the calcaneus distally across the osteotomy site. Next, the cuboid was exposed through the distal portion of the incision, and an osteotomy was made in the midportion of the cuboid. A medial incision was made, and a closing wedge of the bone was removed from the plantar surface of the central third of the medial cuneiform. Then, the osteotomy site was closed with plantar flexion and pronation of the forefoot and stabilized with Kirschner wires or staples. The cuneiform wedge was rotated and inserted into the cuboid (Figure 1a, b).

The modified Kidner procedure was then performed through another medial incision. The ossicle was identified and excised together with the entire synchondrosis and any residual navicular prominence. The tendon was completely detached from the TPT insertion site. The plantar medial tuberosity of the navicular was decorticated. Positioning of the anchor was confirmed under fluoroscopy to ensure that the optimal reattachment point was used and that the navicular-cuneiform joint was not violated. The TPT was advanced and attached to the navicular at neutral resting tension, utilizing the two no. 2 braided nonabsorbable sutures attached to the anchor (Super-Revo®, ConMed Linvatec, Largo, USA) (Figure 1c, d). With the deformity corrected, contracture of the Achilles tendon was assessed, and lengthening was performed, if indicated, with the Vulpius technique (four of 21 feet). The ankle was immobilized in a well-padded short leg cast for 6 weeks. At 6 weeks, patients were allowed to progress to protected weight bearing as tolerated, and physical therapy was initiated for TPT strengthening and gait training in a sneaker. At 12 weeks, patients were transitioned to a normal shoe, and no further bracing or orthotic inserts were usually needed.

### Radiologic parameters

Preoperative and final postoperative follow-up weight-bearing AP and lateral radiographs of the foot were available for all cases. The standing AP radiographs were used to calculate the following four measurements: talo-calcaneal angle, talo-first metatarsal (MTT) angle, talo-fifth MTT angle, and talo-navicular coverage. The standing lateral foot radiographs were used to calculate the following eight measurements: talo-calcaneal angle, talo-first MTT angle, talo-horizontal angle, calcaneal pitch, calcaneo-fifth MTT angle, first-fifth MTT angle, naviculo-cuboid overlap, and medial-lateral column ratio. All radiographic measurements were performed as in Davids et al. [13] and Moraleda et al. [10].

Three orthopedic surgeons (CIP, YJM, SIW) assessed the interobserver reliability of the radiographic measurements. Each examiner took the measurements independently, without knowledge of the patients’ clinical information and the other orthopedic surgeons’ measurements. All measurements were collected by a research assistant (LZ) who did not otherwise participate in this study.

### Clinical parameters

Clinical outcomes were assessed with the American College of Foot and Ankle Surgeons (ACFAS) scoring system preoperatively and at the time of final follow-up. The flatfoot module of the ACFAS score includes both subjective and objective assessment [14]. This system consists of three components: pain (30 points), appearance (5 points), and functional capacity (15 points). A survey was given to each patient at the beginning of the interview. As part of the objective assessment, we determined the range of motion of the ankle and subtalar joints, single-limb heel rise capacity, and limp due to foot pain. The ACFAS questionnaire was scored according to the ACFAS instructional guide [14]. Information was also gathered on alignment of the forefoot, midfoot, and hindfoot; skin breakdown; sports activities; and complications. Patients were evaluated by an independent pediatric orthopedic surgeon who was not involved in their treatment.

### Statistical analysis

Paired *t*-tests were used for the statistical analysis. All analyses were performed with SPSS version 21.0 for Windows (SPSS, Inc., Chicago, IL, USA). *p* values <0.05 were considered statistically significant.

## Results

With the exception of the medial-lateral column ratio, all radiographic measurements showed good to excellent interobserver reliability (Table 1).

**Table 1:** **Interobserver reliability of the radiographic measurements**

**Radiographic measurement**	**ICC**	**95% CI**
AP talo-first MTT angle (°)	0.91	0.81–0.96
AP talo-navicular coverage angle (°)	0.85	0.70–0.94
AP talo-calcaneal angle (°)	0.91	0.81–0.96
AP talo-fifth MTT angle (°)	0.88	0.74–0.95
Lateral talo-first MTT angle (°)	0.89	0.78–0.95
Lateral calcaneal pitch (°)	0.73	0.44–0.88
Lateral calcaneo-fifth MTT angle (°)	0.78	0.55–0.91
Lateral talo-calcaneal angle (°)	0.96	0.93–0.99
Lateral talo-horizontal angle (°)	0.95	0.90–0.98
Lateral first-fifth MTT angle (°)	0.96	0.92–0.98
Lateral naviculo-cuboid overlap (%)	0.89	0.77–0.95
Lateral column ratio (%)	0.58	0.14–0.82

*ICC* intraclass correlation coefficient, *95% CI* 95% confidence interval.

Correction of preoperative deformity was achieved to the normal range in 78% of patients for AP talo-first MTT angle, 69% for AP talo-navicular coverage angle, 91% for talo-calcaneal angle, 46% for AP talo-fifth MTT angle, 70% for lateral talus-first MTT angle, 72% for calcaneal pitch angle, 67% for lateral talo-calcaneal angle, 74% for lateral talo-horizontal angle, 63% for lateral naviculo-cuboid overlap, 45% for lateral calcaneo-fifth MTT angle, and 47% for lateral first-fifth MTT angle. A comparison of the preoperative and final follow-up weight-bearing radiographs showed significant improvement in eight of the 12 radiographic measurements: AP talo-first MTT angle, AP talo-navicular coverage angle, AP talo-calcaneal angle, lateral talo-first MTT, calcaneal pitch, lateral talo-calcaneal angle, lateral talo-horizontal angle, and lateral naviculo-cuboid overlap. We also found marginally significant improvements in three of the 12 radiographic measurements: AP talo-fifth MTT angle, lateral calcaneo-fifth MTT angle, and lateral first-fifth MTT angle (Table 2). The medial-lateral column ratio was significantly decreased from 96.8° (±4.7°) preoperatively to 94.6° (±5.2°) postoperatively (*p* = 0.015). However, this measurement was not useful because interobserver reliability was 0.58 (95% confidence interval (CI), 0.14–0.82). The average ACFAS score improved significantly from 26.1 (±6.8) preoperatively to 44.8 (±5.7) postoperatively (*p* < 0.001) (Table 3).

**Table 2:** **Improvement in radiographic measurements after triple C and the modified Kidner operation**

	**Preoperative**	**Postoperative**	**Mean difference**	***p*** **value** ^**a**^
	**(** ***n*** **= 21)**	**(** ***n*** **= 21)**	**(** ***n*** **= 21)**	
AP talo-first MTT angle (°)	23.3 (4.3)	7.8 (3.1)	15.5 (5.4)	<0.001
AP talo-navicular coverage angle (°)	32.9 (5.1)	11.6 (3.8)	21.3 (5.0)	<0.001
AP talo-calcaneal angle (°)	35.4 (6.1)	18.4 (5.3)	17.0 (6.9)	<0.001
AP talo-fifth MTT angle (°)	33.0 (6.8)	29.2 (6.2)	3.8 (9.9)	0.095
Lateral talo-first MTT angle (°)	26.7 (5.7)	10.1 (2.5)	16.5 (5.8)	<0.001
Lateral calcaneal pitch (°)	7.5 (1.8)	18.2 (3.6)	−10.6 (3.8)	<0.001
Lateral calcaneo-fifth MTT angle (°)	19.0 (5.0)	21.5 (4.6)	−2.6 (6.9)	0.105
Lateral talo-calcaneal angle (°)	45.8 (6.8)	28.5 (7.8)	17.3 (11.2)	<0.001
Lateral talo-horizontal angle (°)	37.3 (5.3)	22.9 (5.3)	14.4 (7.9)	<0.001
Lateral first-fifth MTT angle (°)	8.1 (2.8)	10.3 (3.9)	−2.2 (4.9)	0.057
Lateral naviculo-cuboid overlap (%)	72.6 (8.3)	44.7 (15.7)	27.9 (16.)	<0.001
Medial-lateral column ratio (%)	96.8 (4.7)	94.6 (5.2)	2.2 (3.8)	0.015

^a^Data analyzed with paired *t*-tests; results are mean (± standard deviation).

**Table 3:** **Improvement in clinical outcome after the triple C and modified Kidner operation**

	**Preoperative**	**Postoperative**	**Mean difference**	***p*** **value** ^**a**^
	**(** ***n*** **= 21)**	**(** ***n*** **= 21)**	**(** ***n*** **= 21)**	
ACFAS scores (subjective)	26.1 (6.8)	44.8 (5.7)	−12.6 (5.4)	<0.001
Pain	13.2 (4.3)	25.8 (4.8)	−3.0 (1.2)	<0.001
Appearance	1.5 (1.0)	4.5 (0.7)	−3.1 (2.0)	<0.001
Functional capacity	11.4 (2.7)	14.5 (1.5)	−18.7 (6.8)	<0.001

^a^Data analyzed with paired *t*-tests; results are mean (± standard deviation).

**Figure 2:**
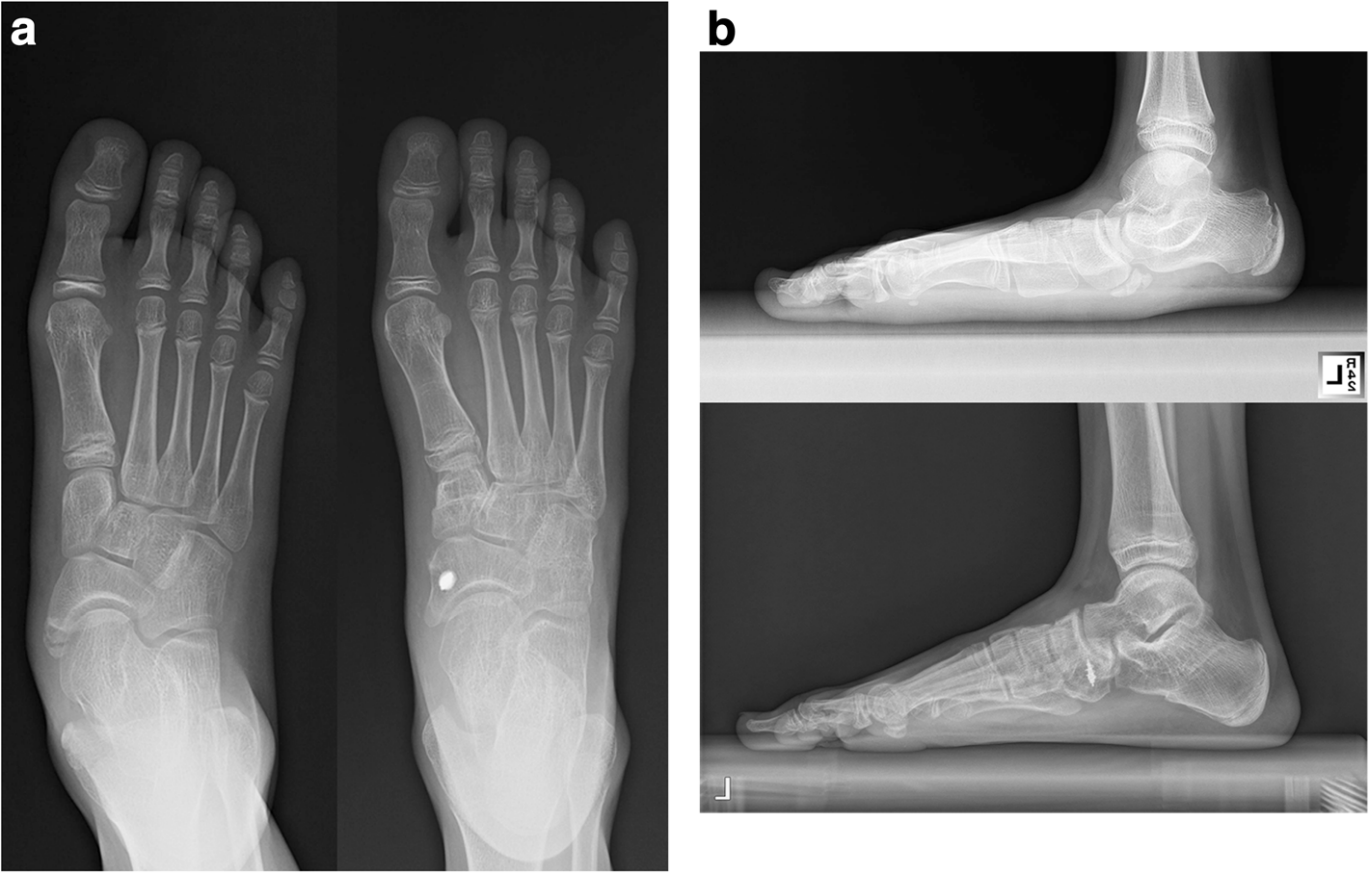
**Comparative preoperative and 1-year postoperative radiographs of a 9-year-old girl. (a)** Preoperative (left) and postoperative (right) weight-bearing AP radiographs demonstrating improvement of AP alignment. **(b)** Preoperative (upper) and postoperative (lower) weight-bearing lateral radiographs showing correction of plantar-flexed talus and restoration of longitudinal arch.

On physical examination, we observed a neutral arch in 17 cases and a flat arch in three cases. There was one varus alignment of the hindfoot due to overcorrection. All forefeet were in a neutral position. There were no wound complications or infections. All patients rated their results as good or excellent. No patients had developed any recurrence of flatfoot deformity either clinically or radiographically at the latest follow-up (Figure 2).

## Discussion

The overall effect of an accessory navicular on foot mechanics is a source of controversy, particularly regarding the association with flatfoot deformity. Flatfoot deformity is present in approximately 50% of patients who become symptomatic, although its role in the development of symptomatic accessory navicular remains unclear [2]. After the synchondrosis was identified as the injured structure in patients who experienced pain, the cause of the trauma became a topic of interest. Tension and shear forces are the direct result of the action of the TPT, whereas compression results from pronation of the subtalar joint in patients who have pes planus.

The initial management of a symptomatic accessory navicular is always conservative. When symptoms become intractable despite adequate conservative treatment, surgical intervention may be indicated. Type II accessory navicular is most likely to require surgical management. The classic Kidner procedure requires excision of the ossicle and rerouting of the TPT to the plantar aspect of the navicular in order to restore the TPT’s ability to support the medial arch. Several investigators have questioned the need for detachment of the TPT, opting instead for excision of the ossicle through a split in the tendon that is subsequently repaired [3,15-17]. When the ossicle is resected, the cartilaginous synchondrosis and any remaining navicular prominence should also be removed. However, in children who have associated flatfoot, excision should be accompanied by rerouting and advancement to increase the tension of the tendon and aid in the reconstruction of the medial longitudinal arch. Recently, fusion has been performed between the ossicle and bone to manage recalcitrant type II accessory navicular [18]. The advantages of this procedure are that it relies on bone-to-bone healing rather than tendon-to-bone healing and that it does not further compromise the TPT by shelling out or resecting the accessory bone.

In cases that involve a more severe, yet flexible, deformity, additional correction can be achieved in conjunction with a Kidner-type procedure with the addition of a subtalar arthroereisis [1]. The procedure is extra-articular, and no bony resection or drilling is required. Although the implant of the subtalar joint may loosen with growth and maturation, it can be removed easily without compromising arch morphology. In cases of severe deformity with loss of flexibility, joint-sparing procedures, such as lateral column lengthening and triple C, can be used in conjunction with the TPT advancement procedure to improve the alignment of the medial longitudinal arch and TPT mechanics. Lateral column lengthening is a redirectional osteotomy of the acetabulum pedis that not only corrects subluxation or dislocation of the subtalar joint, but also preserves motion in the subtalar joint. Nevertheless, many investigators have recommended that LCL not be used for the treatment of severe planovalgus foot deformities because it cannot correct varus and abducted forefoot deformities [19-21]. Furthermore, LCL carries the risk of disrupting the articular surface of the subtalar joint [22]. Rathjen and Mubarak introduced the triple C procedure for correction of planovalgus foot deformities with good results compared with arthrodesis or other techniques [8]. Previously, our group reported that triple C can be a more effective treatment option than LCL for severe flatfoot deformities in children. Moreover, bone grafting is not necessary with this procedure. The mechanism of action of triple C is as follows. First, to address a valgus deformity of the heel, medial shifting of the mechanical pull of the tendon will eliminate the negative effect of the Achilles tendon on the flatfoot deformity. Second, to address forefoot deformity, a cuneiform plantar closing wedge osteotomy will restore the normal talar-first metatarsal angle. Finally, to address the shortening of the lateral column, a cuboid opening wedge osteotomy lengthens the lateral column of the foot. All three procedures help to realign the forefoot and hindfoot.

Many investigators have noted that radiographic measurements are related to clinical outcomes because they provide reliable information on the relationships of different parts of the hindfoot [13,23]. Recently, the reliability and validity of many foot radiographic measurements have been studied by Lee et al. [24], who suggested that naviculo-cuboid overlap, AP talo-navicular coverage angle, and AP talus-first MTT angle are reliable and valid measures for the evaluation of hindfoot valgus and varus deformities. In the present study, eight of 12 radiologic measurements—the AP talo-first MTT angle, AP talo-navicular coverage angle, AP talo-calcaneal angle, lateral talo-first MTT angle, lateral calcaneal pitch, lateral talo-calcaneal angle, lateral talo-horizontal angle, and lateral naviculo-cuboid overlap—improved significantly after surgery. In addition, three other radiologic measurements—the AP talo-fifth MTT angle, lateral calcaneo-fifth MTT angle, and lateral first-fifth MTT angle—were marginally significantly improved. Only the medial-lateral column ratio did not have a satisfactory intraclass correlation coefficient (ICC = 0.58, 95% CI = 0.14–0.82), so this measurement was not analyzed further. Despite the one excluded measurement, the overall improvement in the radiologic measurements was indicative of a satisfactory outcome. In addition, all clinical parameters were significantly improved after surgery. Rathjen and Mubarak considered alignment and patient complaints when assessing the clinical outcomes of the triple C procedure. They obtained good or excellent clinical results in 23 feet (95.8%). In our study, all patients rated their results as good or excellent, and none had developed recurrence of flatfoot deformity either clinically or radiographically at the most recent follow-up, although one patient had varus alignment of the hindfoot. We believe that this complication was caused by overcorrection of the calcaneal osteotomy. Therefore, a careful surgical technique should be used to avoid over- or undercorrection. We assessed the clinical results both subjectively and objectively using the ACFAS score. The ACFAS score improved from 26.1 (±6.8) to 44.8 (±5.7). Pain improved from 13.2 (±4.3) to 25.8 (±4.8), appearance improved from 1.5 (±1.0) to 4.5 (±0.7), and functional capacity improved from 11.4 (±2.7) to 14.5 (±1.5). These results are similar to those of a previous study [8].

Our study had some limitations. First, we included only a small number of patients with a short follow-up period and no control group. Second, the inclusion of unilateral and bilateral cases in the same group could violate the principle of statistical independence. Nevertheless, the study population was homogeneous in terms of the etiology of the condition. Our results have demonstrated that combining triple C with the modified Kidner procedure can correct both the painful prominence and flatfoot deformity. These procedures lead to an appropriate length of the otherwise healthy TPT, and the TPT is able to support the arch, as in a person with normal arch mechanics. Therefore, triple C combined with the modified Kidner procedure can be an effective treatment option for severe flatfoot deformity associated with a symptomatic accessory navicular in children and adolescents.

## Conclusions

Triple C combined with the modified Kidner procedure can be an effective treatment option for severe flatfoot deformity associated with a symptomatic accessory navicular in children and adolescents.
